# Subchondral bone remodeling patterns in larger animal models of meniscal injuries inducing knee osteoarthritis – a systematic review

**DOI:** 10.1007/s00167-023-07579-6

**Published:** 2023-09-24

**Authors:** Tamás Oláh, Magali Cucchiarini, Henning Madry

**Affiliations:** Center of Experimental Orthopaedics, Saarland University, Kirrberger Straße, Building 37, 66421 Homburg/Saar, Germany

**Keywords:** Meniscus, Meniscal tear, Subchondral bone, Animal model, Osteoarthritis, Systematic review

## Abstract

**Purpose:**

Elucidating subchondral bone remodeling in preclinical models of traumatic meniscus injury may address clinically relevant questions about determinants of knee osteoarthritis (OA).

**Methods:**

Studies on subchondral bone remodeling in larger animal models applying meniscal injuries as standardizing entity were systematically analyzed. Of the identified 5367 papers reporting total or partial meniscectomy, meniscal transection or destabilization, 0.4% (in guinea pigs, rabbits, dogs, minipigs, sheep) remained eligible.

**Results:**

Only early or mid-term time points were available. Larger joint sizes allow reporting higher topographical details. The most frequently reported parameters were BV/TV (61%), BMD (41%), osteophytes (41%) and subchondral bone plate thickness (39%). Subchondral bone plate microstructure is not comprehensively, subarticular spongiosa microstructure is well characterized. The subarticular spongiosa is altered shortly before the subchondral bone plate. These early changes involve degradation of subarticular trabecular elements, reduction of their number, loss of bone volume and reduced mineralization. Soon thereafter, the previously normal subchondral bone plate becomes thicker. Its porosity first increases, then decreases.

**Conclusion:**

The specific human topographical pattern of a thinner subchondral bone plate in the region below both menisci is present solely in the larger species (partly in rabbits), but absent in rodents, an important fact to consider when designing animal studies examining subchondral consequences of meniscus damage. Large animal models are capable of providing high topographical detail, suggesting that they may represent suitable study systems reflecting the clinical complexities. For advanced OA, significant gaps of knowledge exist. Future investigations assessing the subchondral bone in a standardized fashion are warranted.

## Introduction

Understanding the spatio-temporal trajectory of subchondral bone remodeling may provide better insights into osteoarthritis (OA) [27]. In the knee, both menisci complement and protect the osteochondral unit [25]. Meniscus injury is common [20, 32], and meniscus tissue insufficiency and loss are one of the most important causes of knee OA [5–7, 21, 49, 52]. The principle of inducing a defined meniscal lesion is applied in many animal models to model OA initiation and development [42, 50]. However, the resulting subchondral bone alterations are incompletely understood [36], in contrast to the cartilage [35]. Though rodents represent by far the most popular model, their small joints limit an accurate topographical recapitulation. Therefore, this systematic review focuses on larger animal models only, analyzing the available knowledge about subchondral bone remodeling applying traumatic meniscal injuries as a standardizing entity. It reports study designs, establishes missing gaps and pinpoints future research directions.

### Comparative morphology of the subchondral bone

As its two major parts, the subchondral bone plate and subarticular spongiosa are structurally dissimilar, they need to be considered separately [37]. The human subchondral bone plate is composed of 0.2–0.4 mm thick plates joining together, enclosing pores, extensions of the marrow space and invading vascular channels [16]. Humans have the largest tibial plateau width (~ 7.4 ± 0.5 cm), followed by sheep (~ 5.1 ± 0.1 cm), minipigs (~ 3.9 ± 0.1 cm), rabbits (~ 1.6 ± 0.1 cm), rats (~ 0.7 ± 0.1 cm), and mice (~ 0.3 ± 0.1 cm). Remarkably, the human subchondral bone plate is considerably thinner (0.52 ± 0.11 mm) than in sheep (1.32 ± 0.14 mm), minipigs (0.82 ± 0.17 mm) and similar to rabbits (0.49 ± 0.05 mm) [39]. In these animals, the subchondral bone plate is more compact and less porous [39]. When normalized to the tibial plateau width, the subchondral bone plate in minipig is twofold, and in sheep and rabbits threefold thicker than in humans [39]. Its microstructure (Table 1) relates to the severity of OA [24, 26, 34, 40]. The subchondral bone plate of rabbits and minipigs is most similar to humans, including similar BS/BV, BS/TV, and closed porosity, while BV/TV is higher, and open and total porosity are lower [39]. In sheep, BV/TV is higher, and BS/BV, BS/TV, open and total porosity are lower than in humans [39]. Of note, the specific human topographical pattern of a thinner subchondral bone plate in the region below both menisci is present solely in the larger species (partly in rabbits), but absent in rodents, a clinically highly relevant fact to consider when designing animal studies examining structural (subchondral) consequences of meniscus damage.

**Table 1:** Bone microstructural parameters frequently used in micro-CT examinations

Name	Abbreviations	Definitions
Percent bone volume	BV/TV	Relative volume of calcified tissue in the selected volume of interest
Bone surface-to-volume ratio	BS/BV	A measure for the bone surface per given bone volume
Bone surface density	BS/TV	Ratio of surface area to total volume
Trabecular thickness	Tb.Th	Thickness of the trabecular structure
Trabecular separation	Tb.Sp	Thickness of the spaces between the trabeculae
Trabecular number	Tb.N	Inverse of the mean distance between the mid-axes of the examined structure
Trabecular pattern factor	Tb.Pf	A parameter of cancellous bone connectivity
Structure model index	SMI	Shows the relative prevalence of plates and rods
Degree of anisotropy	DA	A measure of how highly oriented substructures are within a volume
Fractal dimension	FD	An indicator of surface complexity
Connectivity density	Conn.Dn	Characterizes the redundancy of trabecular connections
Bone mineral density or tissue mineral density	BMD or TMD	Reflects the calcium-hydroxyapatite content

The more porous subarticular spongiosa of sheep, minipigs and rabbits is also more dense and complex than humans, reflected by higher BV/TV, Tb.N, BS/TV, and lower Tb.Sp [39]. For example, the BV/TV of sheep, minipig and rabbit is ~ twofold higher, the Tb.N of sheep and rabbit is ~ twofold, minipig is ~ threefold higher, and the Tb.Sp of sheep is 0.3-fold, minipig is 0.15-fold, and rabbit is 0.4-fold lower than in humans [39]. Structural differences between the lateral and medial tibial plateau trabecular structure as in humans exist in sheep and minipigs [39], but are largely absent in rabbits [39]. These differences include in the medial subregions higher BV/TV (humans, sheep, minipigs), BS/TV (sheep), Tb.Th (humans, minipigs, rabbits), Tb.N, DA and Conn.Dn (sheep), and lower Tb.Pf (humans, sheep), and Tb.Sp (humans) compared to lateral [39]. In contrast, a weaker medial trabecular structure is reflected in a lower BS/BV (humans, minipigs), BS/TV (minipigs), Tb.N (rabbits), FD (sheep, minipigs), Conn.Dn (minipigs), and higher SMI (rabbits), and Tb.Sp (sheep) in the medial subregions compared to lateral [39].

### Pattern of subchondral bone changes

Structural subchondral bone alterations are important characteristics of both early and advanced stages of OA. At onset, primary osteoporotic changes occur [10], besides early degenerative cartilage changes [15, 25, 36, 43, 45]. Subchondral bone plate porosity increases, and BMD, trabecular volume, and complexity of the trabecular structure decrease [15, 43]. These changes may be caused by microdamage of the trabeculae due to altered load [17, 28].

After the initial bone loss, still in early OA, subchondral sclerosis (increase in trabecular volume and complexity), as well as osteophytes occur [25, 43]. Later, pronounced abnormalities of bone shape and cysts appear [25].

### Literature search results

#### (a) PubMed search

**Fig. 1:**
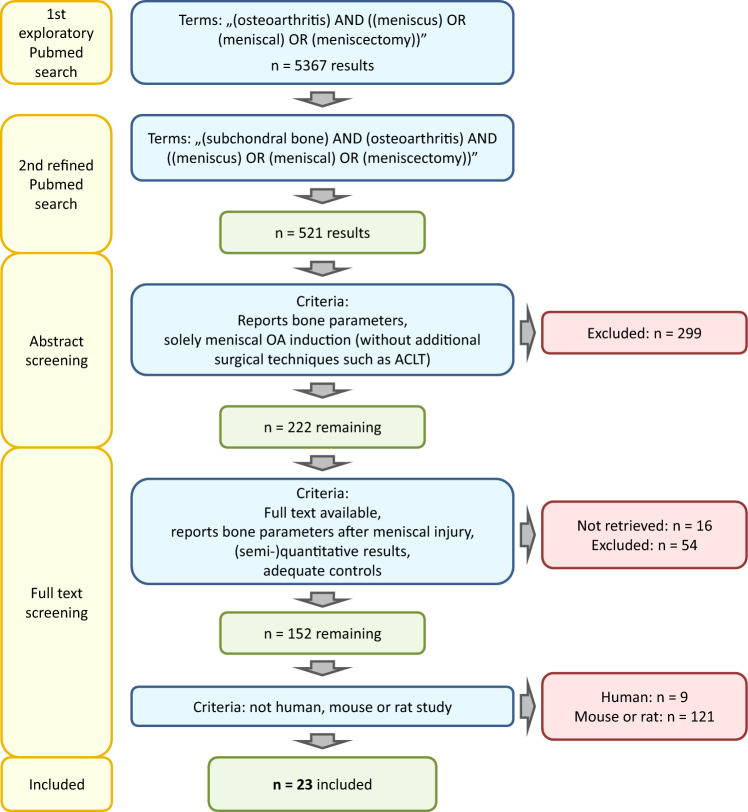
Flowchart of the systematic literature search resulting in n = 23 eligible papers, evaluated in the study

An initial PubMed search was performed on 15.04.2023 with the terms “(osteoarthritis) AND ((meniscus) OR (meniscal) OR (meniscectomy))” yielded 5367 results, including several studies not reporting any subchondral bone data (Fig. 1). When the search was refined as “(subchondral bone) AND (osteoarthritis) AND ((meniscus) OR (meniscal) OR (meniscectomy))”, it resulted in 521 papers (9.7% of the original search). In the detailed analysis only those studies were included where OA was evoked by traumatic tear or injury of the meniscus, surgical destabilization of the meniscus (DMM), or total or partial meniscectomy. To avoid any supplementary factors leading to additional instability such as ligament transection (e.g. anterior cruciate ligament), such combined methods were excluded (Fig. 1). Papers were also excluded if full text was unavailable, if they were reviews, not in English language, subchondral bone or appropriate controls not reported, or meniscal injury not applied to induce OA. The paper selection consequently was further reduced after reading their abstracts (n = 222), and full text (n = 152 papers). When human (n = 9), mouse (n = 96) and rat (n = 25) studies were excluded, n = 23 studies remained eligible for detailed systematic evaluation (only 0.4% of all papers reporting meniscal injury).

#### (b) Evaluation of the abundance of studies

**Fig. 2:**
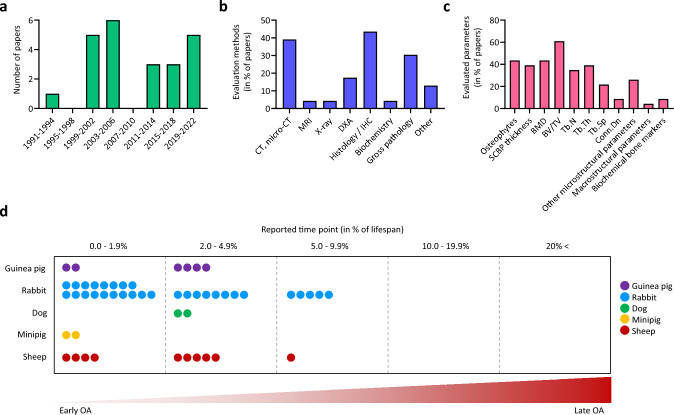
Summary of the n = 23 papers evaluating the subchondral bone in animal models of OA induced by meniscal injuries. **a** Histogram showing the number of eligible papers in 4-year intervals and the most important subchondral bone **b** evaluation methods and **c** parameters most frequently reported. Note that the cumulative percentages within the graphs may not be equal to 100% due to some studies reporting multiple techniques or parameters. **d** Reported time points in all of the studies (total n = 23), expressed as percentage of the average life span [41, 42] of the species. Dots indicate study termination time points corresponding to the displayed percent range. Papers reporting multiple time points are presented with multiple dots on the figure. *CT* computed tomography; *DXA* Dual Energy X-ray Absorptiometry; *IHC* immunohistochemistry; *MRI* magnetic resonance imaging; *SCBP* subchondral bone plate

The first animal study fulfilling the inclusion criteria was published in 1993 [3]. The abundance of eligible papers was constantly low afterwards (n = 0–6 in 4-year intervals) (Fig. 2a).

#### (c) Evaluation of the study designs

In the finally selected 23 papers, four main types of induced meniscal damage were described: (1) total meniscectomy, (2) partial meniscectomy, (3) meniscal transection or tear (MMT; the medial collateral ligament [MCL] is transected in small animals and left intact in large ones, and the pars intermedia is transected at its narrowest point, but no parts of the meniscus are removed), and (4) DMM by “meniscal release” (the anterior root is transected, but no parts are removed).

#### (d) Evaluation of study methods

The most common method to evaluate subchondral bone structure was histology, used in 44% of the studies (n = 10). Micro-CT was used only in 39% of all studies (n = 9), although it represents the gold-standard method for directly evaluating bone microstructure [9], with excellent reproducibility and accuracy [8] (Fig. 2b). When the occurrence of applying a combined evaluation protocol including microcomputed-tomography (micro-CT) or histology, paired with dual energy X-ray absorptiometry (DXA), biochemistry or gross pathology of the joint was examined, histological evaluation was mostly used alone (40% of the total n = 23 studies), or in combination with gross pathology (30%), or DXA (30%). Micro-CT was used alone in 30% of all studies, and in combination with gross pathology in 9%. Histological evaluation was mostly applied for reporting subchondral bone plate thickness, but several studies also used it to evaluate subchondral trabecular microstructure, despite the strong limitation of the stereologic analysis of a few 2-dimensional (2D) sections assuming plate-like underlying structure [8]. Many of such studies did not identify significant differences between treatment groups [23, 33]. Overall, the most frequently reported bone parameters were BV/TV, BMD, presence of osteophytes, Tb.Th, thickness of the subchondral bone plate, and Tb.N (Fig. 2c). Larger animal studies only reported early and mid-term time points (Fig. 2d).

### Subchondral bone changes caused by meniscus damage in different larger animal models

#### (a) Guinea pigs

Partial medial meniscectomy and “meniscectomy” (with unclear definition, probably rather involving MMT) protocols were used in four studies (Table 2). In the partial medial meniscectomy model, at 1 month, BMD decreased [47], and subchondral bone plate thickness [46] and BV [46, 47] were unchanged. At 3 months, subchondral bone plate thickness [46] and medial BMD [47] increased (BV unchanged), indicating subchondral bone plate sclerosis at mid-term. In a (probably) MMT model, at 12 weeks, osteophytes [18], decreased trabecular BMD [18, 19], BV/TV [18, 19], Tb.Th [18, 19], and increased Tb.Sp [18, 19], SMI [18, 19], and Tb.Pf [19] were reported, indicating a loss of trabecular bone. Of note, in the Hartley guinea pigs, a commonly used outbred strain of short haired albino guinea pigs, the usage of appropriate, age-matched, sham operated controls is exceedingly important due to the commonness of spontaneous OA.

**Table 2:** Studies describing OA alterations of the guinea pig subchondral bone following surgically induced meniscal injuries

Meniscal damage	Study design	Follow-up duration	Used methods, evaluated subchondral bone parameters	Relevant findings in OA	References
Partial medial meniscectomy	Unilateral: (1) left partial meniscectomy + right unoperated; (2) left sham + right unoperated	1, 3 months	Histology: subchondral bone plate + subchondral trabecular bone: bone volume %; DXA: BMD	1 month: no difference in bone volume, lower BMD than in sham or in contralateral 3 months: higher bone volume than in contralateral unoperated, and no difference vs. sham. Higher medial BMD than in contralateral unoperated	[47] Pastoureau 1999
Partial medial meniscectomy	Unilateral: (1) left partial meniscectomy + right unoperated; (2) left sham + right unoperated	1, 3 months	Histology: subchondral bone plate thickness; subchondral bone plate + subchondral trabecular bone: bone volume %	At 1 month, no difference in subchondral bone plate thickness, at 3 months it increased. Bone volume did not differ vs. sham (but it increased in sham from 1 to 3 months)	[46] Pastoureau 2003
MMT (+ MCLT) (?) *(written as medial meniscectomy; unclear protocol; rather MMT)*	Laterality not reported: (1) MMT (?) (2) sham (3) other treatment group	12 weeks	Micro-CT: subchondral trabecular bone: BMD, BV/TV, Tb.N, Tb.Sp, Tb.Th, SMI, DA; gross pathology: osteophytes	Macroscopic and histological cartilage scores were worse, cartilage thickness decreased, subchondral trabecular bone BMD, BV/TV, Tb.Th decreased, Tb.Sp, SMI, Tb.Pf increased compared to sham	[19] Dai 2016
MMT (+ MCLT) (?) *(written as medial meniscectomy; unclear protocol; rather MMT)*	Unilateral: (1) left unoperated + right MMT (?); (2) left unoperated + right sham; (3) other treatment group	12 weeks	Micro-CT: subchondral trabecular bone: BMD, BV/TV, Tb.N, Tb.Sp, Tb.Th, SMI, DA; gross pathology: osteophytes	Macroscopic and histological cartilage scores were worse, cartilage thickness decreased, osteophytes were found, and subchondral trabecular bone BMD, BV/TV, Tb.Th decreased, Tb.Sp, SMI increased compared to sham	[18] Chu 2017

At the study design “other treatment groups” mean medical or surgical treatments irrelevant for the present review

*BMD* bone mineral density; *BV/TV* percent bone volume; *DA* degree of anisotropy; *DXA* Dual Energy X-ray Absorptiometry; *MCLT* medial collateral ligament transection; *MMT* medial meniscal transection; *SMI* structure model index; *Tb.N* trabecular number; *Tb.Sp* trabecular separation; *Tb.Th* trabecular thickness

**Fig. 3:**
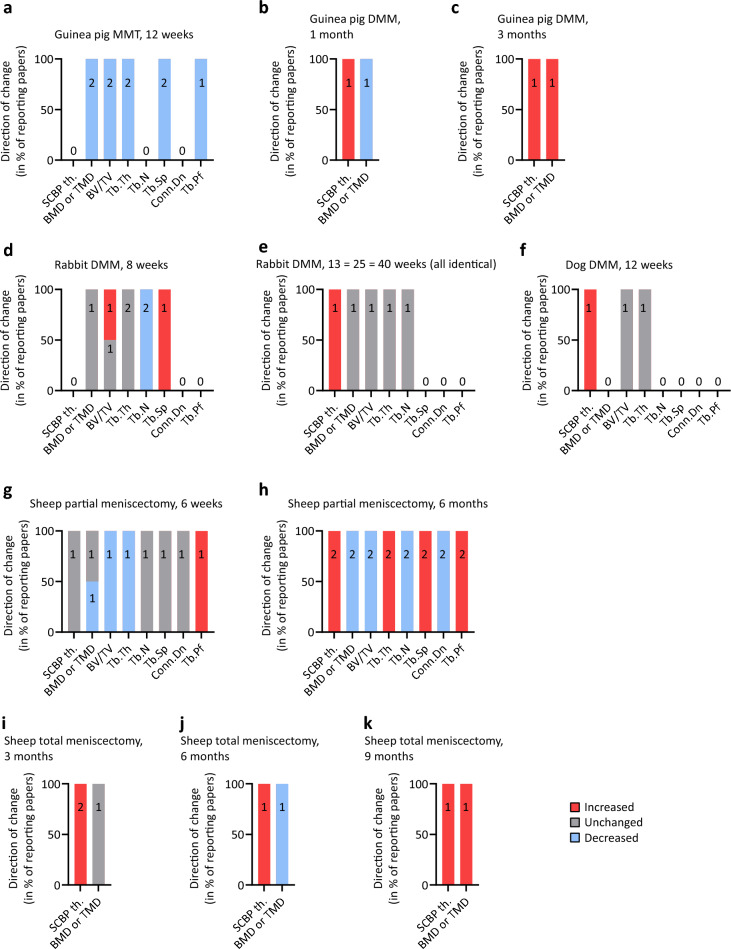
Numbers and ratios of studies reporting the directions of changes of the individual bone microstructural parameters at different time points in multiple species. Stacked column diagrams showing OA-related changes of the bone microstructural parameters following **a** guinea pig medial meniscal transection (MMT) at 12 weeks and guinea pig destabilization of the medial meniscus (DMM) at **b** 1 and **c** 3 months, rabbit DMM at **d** 8, and at **e** 13–40 weeks, **f** dog DMM at 12 weeks, and sheep partial meniscectomy at **g** 6 weeks and **h** 6 months, and total meniscectomy at (**i**) 3, (**j**) 6, (**k**) 9 months. Numbers in columns show the number of studies evaluated. *BMD or TMD* bone or tissue mineral density; *BV/TV* percent bone volume; *Conn.Dn* connectivity density; *SCBP th.* subchondral bone plate thickness; *Tb.N* trabecular number; *Tb.Pf* trabecular pattern factor; *Tb.Sp* trabecular separation; *Tb.Th* trabecular thickness

In sum, meniscal damage at 3 months in guinea pigs resulted in increased subchondral bone plate thickness and loss of trabecular bone (Table 2, Fig. 3a–c), similarly to other early/mid-term OA models.

#### (b) Rabbits

Studies examining the consequences of total and partial medial meniscectomy, or of anterior medial or lateral root tears did not report major subchondral bone changes (Table 3, Fig. 3d, e). At 2 weeks after total meniscectomy, subchondral bone plate BMD decreased [1]. At 3 weeks, a bone remodelling score was unchanged, scintimetric uptake increased [22]. Between 3 and 8 weeks, regional bone blood flow (tibial plateau, femoral condyles) increased [2]. At 4 and 8 weeks, subchondral bone plate BMD decreased [1].

**Table 3:** Studies describing OA alterations of the rabbit subchondral bone following surgically induced meniscal injuries

Meniscal damage	Study design	Follow-up duration	Used methods, evaluated subchondral bone parameters	Relevant findings in OA	References
Total medial meniscectomy	Bilateral: (1) left sham + right meniscectomy (2) left unoperated + right unoperated	13, 25, 40 weeks	DXA: whole knee and subregional BMD; gross pathology: osteophytes	Cartilage fibrillation and osteophytes after meniscectomy. No difference in overall or regional BMD between sham and meniscectomy, but on frontal projections both had lower BMD than unoperated controls. No regrowth of the meniscus	[38] Messner 2000
Medial meniscectomy	Bilateral: (1) left sham + right meniscectomy (2) left unoperated + right unoperated	13, 25, 40 weeks	Histology: subchondral bone plate: thickness; subchondral bone plate + subchondral trabecular bone: BV/TV; subchondral trabecular bone: BV/TV, BS/TV, BS/BV, Tb.Th, Tb.N	Increased subchondral bone plate thickness in the peripheral MTP, and with follow-up time. Subchondral bone plate + subchondral trabecular bone BV/TV and subchondral trabecular bone structure did not differ from sham and unoperated	[23] Fahlgren 2003
Total medial meniscectomy	Unilateral: left unoperated + right meniscectomy	Weekly at 0–11 weeks	Fluorescent microsphere method: regional bone blood flow	At 3–8 weeks regional bone blood flow increased in the tibial plateau and femoral condyles, but not in other bone regions	[2] Anetzberger 2004
Total medial meniscectomy (with capsular incision)	Bilateral: (1) right mensicectomy + left capsular incision (2) left unoperated + right unoperated	3, 6, 12 weeks	Histology: bone remodelling score; bone scintimetry; mmunohistochemistry: osteogenic protein (OP-1)	At 3 weeks severe cartilage changes, higher presence of OP-1 in cartilage, higher scintimetric uptake than at 6 weeks and higher than in control. No significant difference in bone remodelling score	[22] Fahlgren 2006
Total medial meniscectomy	Unilateral: (1) left unoperated + right meniscectomy (2) sham (no details available)	2, 4, 8, 12, 24 weeks	CT osteoabsorptiometry: subchondral bone plate BMD	Worsening macroscopic cartilage damage over time. Subchondral bone plate BMD decreased at 2–8 weeks, and then became normal at 12–24 weeks. Subchondral bone plate BMD density maximum area at the anterior position shifted more to the margin of the MTP with time	[1] Anetzberger 2014
Partial medial meniscectomy (anterior half)	Unilateral: (1) left partial meniscectomy + right unoperated (2) left sham + right unoperated; (3) left unoperated + right unoperated	2, 4, 6, 8, 10 weeks	MRI and X-rays: sclerosis, cysts, osteophytes; X-rays: Tb.N (number of trabecules vs total volume), bone density (percentage of trabecular bone vs total volume), mean trabecular size (bone trabecular density vs trabecular number), and volume fraction	No cysts, osteophytes or sclerosis were identified with MRI. Cartilage thickness at the weight-bearing area of the femur progressively increased at 4–8 weeks. Medial but not lateral osteophytes, discoloration of the cartilage at later time points. X-rays and bone structure did not differ at any time point	[14] Calvo 2001
DMM	Unilateral: (1) left unoperated + right DMM (2) left unoperated + right sham (3) other treatment groups	8 weeks	Micro-CT: BV/TV, Tb.Th, Tb.N	BV/TV increased, Tb.N decreased, Tb.Th did not change	[53] Zhou 2021
DMM (posterior root released)	Laterality not reported: (1) DMM (2) sham (3) other treatment groups	12 weeks	Micro-CT (whole subchondral bone): BV/TV	BV/TV increased	[51] Yan 2021
(1) DMM by anterolateral meniscal root tears; (2) DMM by anteromedial meniscal root tears	Unilateral: (1) left or right (alternating) anterolateral meniscal root tear + contralateral unoperated (2) left or right (alternating) anteromedial meniscal root tear + contralateral unoperated	8 weeks	Micro-CT: subchondral bone plate: BMD, BV/TV; subchondral trabecular bone: BMD, BV/TV, Tb.N, Tb.Th, Tb.Sp	After anterolateral meniscal root tears the medial uncovered Tb.Sp increased, Tb.N decreased. After DMM by anteromedial meniscal root tears the lateral uncovered Tb.Sp increased, Tb.N decreased. No significant changes were observed in the cartilage and in any other subregions in any other bone parameters (subchondral trabecular bone BMD, Tb.Th, BV/TV, and subchondral bone plate BMD, BV/TV)	[48] Steineman 2017

*BMD* bone mineral density; *BS/BV* bone surface-to-volume ratio; *BS/TV* bone surface density; *BV/TV* percent bone volume; *CT* computed tomography; *DMM* destabilization of the medial meniscus; *DXA* Dual Energy X-ray Absorptiometry; *Tb.N* trabecular number; *Tb.Sp* trabecular separation; *Tb.Th* trabecular thickness

In contrast, beginning at 12 weeks, subchondral bone plate BMD [1] did not differ from sham operated knees. At 13 weeks, osteophyte developed [38] and the subchondral bone plate became thicker (peripheral regions, medial tibial plateau) [23], while BMD [38], total subchondral bone and trabecular BV/TV [23], trabecular BS/TV [23], BS/BV [23], Tb.Th [23], and Tb.N [23] were unchanged. At 24 weeks, unchanged subchondral bone plate BMD [1] was found. At 25 and 40 weeks, the bone structure was similar to the 13 weeks time point, with osteophytes [38], increased subchondral bone plate thickness in the peripheral regions of the medial tibial plateau [23], unchanged BMD [38], subchondral bone and trabecular BV/TV [23], trabecular BS/TV [23], BS/BV [23], Tb.Th [23], and Tb.N [23].

In sum, total meniscectomy induced only minor changes in the subchondral bone, including decreased subchondral bone plate BMD and increased bone blood flow at the earlier time points (2–8 weeks). Osteophytes developed in the later phase after 12 weeks, while BMD and trabecular structure were unchanged.

After partial meniscectomy, no cysts, osteophytes, or sclerosis, and unchanged bone structure [14] were reported at 2–10 weeks. After DMM, at 8 weeks, increased [53] or unchanged [48] BV/TV, decreased Tb.N [53], decreased lateral Tb.N after medial meniscal root tear [48], decreased medial Tb.N after lateral root tear [48], unchanged Tb.Th [48, 53], unchanged trabecular and subchondral bone plate BMD [48], increased lateral Tb.Sp after medial root tear [48], and increased medial Tb.Sp after lateral root tear [48], were reported. At 12 weeks, BV/TV increased [51].

In sum, DMM induced only minor changes of the subchondral bone after 8–12 weeks, including increased BV/TV, and trabecular bone loss mostly in the compartment opposing the operated compartment. The fact that no characteristic major microstructural changes were detected (Fig. 3d, e) might be due to the limited sensitivity of the applied 2D detection methods applied. In order to achieve better comparability with human and other animal data, more short- and long-term studies with sensitive 3D detection methods such as micro-CT are needed.

#### (c) Dogs

Partial and total meniscectomy and meniscal destabilization were examined in 2 studies (Table 4, Fig. 3f) [31, 33]. Both partial and total meniscectomy at 16 weeks evoked non-significantly increased percentage bone area, besides largely similar cartilage damage, and evidence of meniscal repair [31]. DMM at 12 weeks resulted in increased medial tibial plateau subchondral bone plate thickness [33], unchanged trabecular BV/TV [33] and Tb.Th [33] besides medial compartment cartilage pathology [33].

**Table 4:** Studies describing OA alterations of the canine subchondral bone following surgically induced meniscal injuries

Meniscal damage	Study design	Follow-up duration	Used methods, evaluated subchondral bone parameters	Relevant findings in OA	References
(1) partial medial meniscectomy (caudal pole hemi-meniscectomy, posterior half of the medial meniscus removed); (2) total medial meniscectomy	Unilateral: (1) left unoperated + right partial meniscectomy; (2) left unoperated + right total meniscectomy	16 weeks	Histology: percentage bone area	Partial and total meniscectomy groups were largely similar, including shifted weight distribution to the control limb, some degree of meniscal regeneration, macroscopic and microscopic cartilage damage in the medial tibial plateau and medial femoral condyle, and slightly but not significantly increased percentage bone area (subchondral bone density)	[31] Johnson 2004
DMM (medial meniscal release by transection of posterior horn)	Unilateral: (2) left unoperated + right sham (3) other treatment groups	12 weeks	Histology: subchondral bone plate: thickness; subchondral trabecular bone: BV/TV, Tb.Th	Medial compartment cartilage pathology, increased medial tibial plateau subchondral bone plate thickness, unchanged trabecular BV/TV and Tb.Th	[33] Kuroki 2011

At the study design “other treatment groups” mean drug or surgical treatments irrelevant for the present review

*BV/TV* percent bone volume; *DMM* destabilization of the medial meniscus; *Tb.Th* trabecular thickness

Thus, data from dogs are scarce and analyses only based on 2D histological sections which have limited accuracy compared to true 3D analyses with micro-CT [8]. Furthermore, time points covered only the 12–16 week period corresponding to early/mid stage OA, when no extensive alterations of the subchondral bone were observed. To allow for a detailed comparison with human and other animal data, more shorter- and longer-term studies are necessary.

#### (d) Minipigs

Only one study described purely meniscus-related OA changes of the subchondral bone [4] (Table 5). In Yucatan minipigs, at 1 month after DMM, cartilage contact area decreased and concentrated at the cartilage-cartilage region [4], deep BV/TV decreased [4], and superficial Tb.Th increased [4]. At 3 months, contact area, deep BV/TV, and superficial Tb.Th all became normal [4]. These changes might be due to an early transient loss of smaller trabeculae caused by increased loads, which is consistent with other early OA models [15].

**Table 5:** Study describing OA alterations of the porcine subchondral bone following surgically induced meniscal injury

Meniscal damage	Study design	Follow-up duration	Used methods, evaluated subchondral bone parameters	Relevant findings in OA	Reference
DMM (transection of the anterior horn of the medial meniscus)	Bilateral: DMM or sham (no further details available)	1 or 3 months	Micro-CT: superficial (subchondral bone plate + subchondral trabecular bone), deep (subchondral trabecular bone): BV/TV, Tb.Th	1 month: contact area decreased and concentrated at the cartilage-cartilage region, deep BV/TV decreased, superficial Tb.Th increased 3 months: contact area, deep BV/TV, superficial Tb.Th returned to baseline (to the same level as sham, which did not change with time)	[4] Bansal 2020

*BV/TV* percent bone volume; *DMM* destabilization of the medial meniscus; *Tb.Th* trabecular thickness

Longer duration studies are needed to examine whether the minipig DMM model shows similar late OA subchondral bone sclerosis as humans.

#### (e) Sheep

A detailed systematic review analyzed meniscectomy-induced OA in sheep [50], although the focus was not on subchondral bone alterations and no studies before 2010 were reported. Surgical OA induction was achieved via total medial (n = 2 studies) [3, 12], total lateral (n = 3 studies) [11, 13, 29], and anterior partial medial (n = 2 studies) [43, 44] meniscectomy, complete transection of the medial pars intermedia (i.e. MMT; n = 1 study) [12], and DMM (n = 1 study) [12] (Table 6).

**Table 6:** Studies describing OA alterations of the ovine subchondral bone following surgically induced meniscal injuries

Meniscal damage	Study design	Follow-up duration	Used methods, evaluated subchondral bone parameters	Relevant findings in OA	References
Total medial meniscectomy	Unilateral: (1) left unoperated + right meniscectomy (2) left unoperated + right unoperated (3) other treatment groups	6 months	Gross pathology: osteophytes	Cartilage damage and osteophytes. Physical exercise exacerbates the OA changes	[3] Armstrong 1993
Total lateral meniscectomy	Bilateral: (1) left meniscectomy + right meniscectomy (2) left unoperated + right unoperated (3) other treatment group	3 and 6 months	Histology: subchondral bone plate thickness Gross pathology: osteophytes	Cartilage erosion, osteophytes, worse Mankin scores at 3 months at lateral, and at 6 months at lateral and medial subregions. Increased subchondral bone plate thickness, cartilage thickness, cartilage area at 6 months in the lateral compartment (data at 3 months not reported)	[13] Cake 2000
Total lateral meniscectomy	Bilateral: (1) left meniscectomy + right meniscectomy (2) left unoperated + right unoperated; (3) other treatment group	3, 9 months	Histology: subchondral bone plate thickness DXA: subchondral bone plate + subchondral trabecular bone: BMD	At 3 and 9 months increased lateral femoral condyle outer region cartilage thickness, macroscopic early OA morphology of the cartilage, lateral osteophytes. Subchondral bone plate thickness increased at the lateral tibial plateau outer region at 3 months, at the lateral femoral condyle at 3 and 9 months. In BMD no difference at 3 months, increase at 9 months at outer and middle lateral tibial plateau and outer lateral femoral condyle	[29] Hwa 2001
Total lateral meniscectomy	Bilateral: (1) left meniscectomy + right meniscectomy (2) left unoperated + right unoperated (3) other treatment group	6 months	DXA: BMD; histology: subchondral bone plate thickness; gross pathology: osteophytes; radioimmunoassay: serum osteocalcin; HPLC: urine pyridinoline and deoxypyridinoline	Macroscopic cartilaginous OA changes and osteophyte development; increased serum osteocalcin, no change in urine pyridinoline and deoxypyridinoline (bone degradation markers), increased medial and decreased lateral BMD, thicker lateral and thinner medial outer subchondral bone plate, worse cartilage histology scores especially at lateral compartment, decreased dynamic shear modulus, decreased inner and increased outer cartilage thickness	[11] Cake 2004
(1) Total medial meniscectomy; (2) MMT (mid-body transection of the medial meniscus); (3) DMM (cranial pole meniscal release)	Unilateral: (1) left unoperated + right meniscectomy (2) left unoperated + right MMT (3) left unoperated + right DMM (4) left unoperated + right sham	12 weeks	Histology: subchondral bone plate thickness gross pathology: osteophytes	Both meniscectomy, MMT and DMM: moderate unloading of the operated knee in gait, largely similar gross pathology, and cartilage histology, worse at medial than lateral, and all worse than sham (no protective effect of the residual meniscal tissue). No change in cartilage thickness DMM: more cranial and focal lesions Meniscectomy and MMT: more widespread lesions, osteophyte formation, gelatinous “meniscoid” regrowth, increased subchondral bone plate thickness in middle zone of medial femoral condyle	[12] Cake 2013
Anterior partial medial meniscectomy	Unilateral: left unoperated + right partial meniscectomy	6 weeks, 6 months	Micro-CT: osteophyte area, subchondral bone plate: thickness, porosity, BMD, BV/TV, BS/BV, BS/TV Subchondral trabecular bone: BMD, BV/TV, BS/BV, BS/TV, Tb.Th, Tb.Sp, Tb.N, Tb.Pf, SMI, DA, FD, Conn.Dn	6 weeks: Anterior subregions: macroscopic and microscopic cartilage damage, small osteophytes, unchanged subchondral bone plate thickness, increased subchondral bone plate porosity, decreased subchondral bone plate BMD, BV/TV; distinct loss of subchondral trabeculae (decreased BV/TV, Tb.Th, increased BS/BV, Tb.Pf, unchanged BS/TV, BMD, Tb.N, Conn.Dn, Tb.Sp, SMI, DA, FD) Other subregions of the medial tibial plateau: minor loss of subchondral trabeculae (decreased BV/TV, Tb.Th, BMD, unchanged BS/TV, Tb.N, Conn.Dn, Tb.Sp, BS/BV, SMI, dA, Tb.Pf, FD), no change at subchondral bone plate 6 months: Anterior subregions: macroscopic and microscopic cartilage damage, increased subchondral bone plate thickness, decreased subchondral bone plate porosity, BS/TV, BS/BV, BMD, disrupted osteochondral correlations, large osteophytes Other subregions: strong loss of subchondral trabeculae (decreased BS/TV, BV/TV, BMD, Tb.N, Conn.Dn, BS/BV, DA, FD increased Tb.Sp, Tb.Th, Tb.Pf)	[43] Oláh 2019
Anterior partial medial meniscectomy	Unilateral: (1) left unoperated + right partial meniscectomy with neutral HTO (2) other treatment groups	6 months	Micro-CT: osteophyte area, subchondral bone plate: thickness, porosity, BMD, BV/TV, BS/BV, BS/TV Subchondral trabecular bone: BMD, BV/TV, BS/BV, BS/TV, Tb.Th, Tb.Sp, Tb.N, Tb.Pf, SMI, DA, FD, Conn.Dn	Anterior subregions: macroscopic and microscopic cartilage damage, decreased subchondral bone plate porosity, BS/TV, BS/BV, BMD, disrupted osteochondral correlations, large osteophytes Other subregions: strong loss of subchondral trabeculae (decreased BS/TV, BV/TV, BMD, Tb.N, Conn.Dn, BS/BV, DA, FD increased Tb.Sp, Tb.Th, Tb.Pf)	[44] Oláh 2022

At the study design “other treatment groups” mean drug or surgical treatments irrelevant for the present review

*BMD* bone mineral density; *BS/BV* bone surface-to-volume ratio; *BS/TV* bone surface density; *BV/TV* percent bone volume; *Conn.Dn* connectivity density; *DA* degree of anisotropy; *DMM* destabilization of the medial meniscus; *DXA* Dual Energy X-ray Absorptiometry; *FD* fractal dimension; *HPLC* High Performance Liquid Chromatography; *MMT* medial meniscal transection; *SMI* structure model index; *Tb.N* trabecular number; *Tb.Pf* trabecular pattern factor; *Tb.Sp* trabecular separation; *Tb.Th* trabecular thickness

After total (medial or lateral) meniscectomy, at 3 months, osteophytes [12, 13, 29], increased subchondral bone plate thickness [12, 29], and unchanged BMD [29] were observed. At 6 months, osteophytes [3, 11, 13], increased subchondral bone plate thickness [13], decreased operated (lateral) and increased contralateral (medial) compartment BMD [11], and thicker operated and thinner contralateral compartment subchondral bone plate [11] were detected. At 9 months, subchondral bone plate thickness [29], BMD [29] increased and osteophytes developed [29]. Thus, osteophytes and increased subchondral bone plate thickness are characteristic of both the relatively early and more advanced stages of OA, while BMD first decreased at mid-term, and then increased at a more advanced stage.

At the anterior subregions after anterior partial medial meniscectomy, at 6 weeks, small osteophytes, increased subchondral bone plate porosity, unchanged subchondral bone plate thickness, decreased subchondral bone plate BMD; distinct loss and thinning of subchondral trabeculae were observed besides developing cartilage damage [43]. In other subregions of the medial tibial plateau, such changes were minor and the subchondral bone plate unchanged [43]. At 6 months, subchondral bone plate thickness increased, its porosity and BMD decreased, and large osteophytes occurred in the anterior subregions. The entire medial tibial plateau exhibited a strong loss of subchondral trabeculae (decreased BMD, and Tb.N, and increased Tb.Sp, and Tb.Th) [43, 44]. These data reveal a progressive loss of subchondral trabeculae, starting below the location of the meniscal injury at early and mid-term OA, reflected in disrupted correlations of microstructural osteochondral parameters.

Total meniscectomy, MMT and DMM all induced largely similar cartilage damage at 3 months [12]. Differences included more anterior and focal cartilage lesions in DMM versus more widespread lesions. Osteophyte formation and subchondral bone plate thickness increased after total meniscectomy and MMT [12].

In sum, early and mid-term ovine OA development was observed in the studied 1.5–9 months’ time-frame. It is characterized by a local deterioration of the subchondral bone with osteophyte development, increased subchondral bone plate thickness, loss of bone volume and trabeculae, and decreased mineralization affecting primarily the compartment with compromised meniscal integrity, mostly independently of the applied technique (Table 6, Fig. 3g–k). Importantly, studies resembling human late OA by examining such ovine subchondral bone changes with longer follow-up time (several years) are noticeably lacking.

## Discussion

The most important finding is the spatio-temporal pattern of subchondral bone remodeling: Changes in the subarticular spongiosa occur shortly before those of the subchondral bone plate. These early alterations involve a degradation of the trabecular elements, reduction of their number, loss of bone volume and reduced mineralization. Soon thereafter, the previously normal subchondral bone plate becomes thicker. Its porosity first increases, then decreases. Other essential conclusions are that: (1) Only early or mid-term time points were presented. (2) Larger joint sizes allow reporting higher topographical details. (3) The most frequently reported bone parameters were BV/TV (61%), BMD (41%), osteophytes (41%) and subchondral bone plate thickness (39%). (4) Subchondral bone plate microstructure is not comprehensively characterized. (5) Microstructure of the subarticular spongiosa is well described.

Out of the 5367 identified meniscus-related OA studies, only 521 (9.7%) mentioned subchondral bone in its title or abstract, and out of them only 23 (0.4%) fulfilled the criteria to report subchondral bone characteristics in the surgical protocol solely based on meniscal damage in guinea pigs, rabbits, dogs, minipigs, and sheep. Data on dogs and minipigs were scarce with only a few published studies. For dogs, this might be due to the public perception being companion animals, the often complex ethical approval processes, and their difficult and costly management [41]. Minipigs require specialized husbandry and food, and they are considerable less docile than sheep, and the miniature strains, more suitable for OA research than large agricultural pigs, are possibly less widely available in some countries [41]. No data from horses or goats were identified. Some large animal studies reported their results in high topographic details, usually examining only 1 or 2 time points. In rabbits, contrastingly, 3–5 end points, covering a broader time scale from early to mid-term / late OA were sometimes reported. Only a low percentage (0.4%) of studies report subchondral bone characteristics. The number of such studies did not change recently for the examined larger species. Among them, rabbits are most frequently used (39%). OA is induced mostly in the medial compartment (87%), in a unilateral study design (61%), in the right (57%) knee, by total meniscectomy (48%). Micro-CT was only selected in 39% of the studies (histology: in 44%), although it is the most capable and recommended [8] method to analyze bone structure at high detail.

**Fig. 4:**
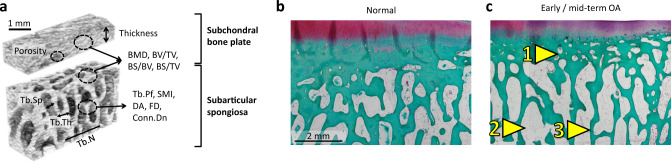
Summary of the reported subchondral bone microstructural changes in early/mid-term OA. **a** 3-dimensional reconstructed micro-CT model of the subchondral bone plate and subarticular spongiosa showing the generally evaluated microstructural parameters. Representative safranin-O/fast-green stained histological sections of the medial tibial plateau of **b** a normal sheep and **c** a sheep 6 months after partial medial meniscectomy [43]. Arrowheads point to characteristic subchondral bone microstructural alterations described in multiple animal models in early / mid-term OA, including (1) increased subchondral bone plate porosity, and degradation of the trabecular elements with (2) increased trabecular separation, and (3) reduction of their number, loss of bone volume and reduced mineralization. *BMD* bone mineral density; *BS/BV* bone surface-to-volume ratio; *BS/TV* bone surface density; *BV/TV* percent bone volume; *Conn.Dn* connectivity density; *DA* degree of anisotropy; *FD* fractal dimension; *SMI* structure model index; *Tb.N* trabecular number; *Tb.Pf* trabecular pattern factor; *Tb.Sp* trabecular separation; *Tb.Th* trabecular thickness

Eligible work presented only early or mid-term time points of OA development. Studies reporting more severe damage in the region below a meniscus lesion confirm the spatio-temporal pattern of subchondral bone remodeling induced by a meniscus injury [43, 44]. At the site of the injury, osteophytes appear relatively soon. Remarkably, changes in the subarticular spongiosa appear slightly before subchondral bone plate alterations, as ovine data suggests. These early alterations are characterized by a degradation of the trabecular elements and reduction of their number (decreased Tb.N), loss of bone volume (mostly decreased trabecular BV/TV, increased Tb.Pf and Tb.Sp), and reduced mineralization (BMD, TMD). Soon afterwards, the previously normal subchondral bone plate becomes thicker. Its porosity, a parameter negatively associated with sclerosis, first in-, then decreases (Fig. 4).

Methodological issues were also identified (Table 7). A detailed, quantitative 3D microstructural assessment of the subchondral bone is still not a general practice (performed in ~ 1/5 of the studies), limiting our knowledge about subchondral remodeling. Although cartilage analyses are relatively well standardized, similar methodical guidance of bone evaluation is absent. Such data would be needed to allow for clinically relevant and comparable conclusions in distinct model species and time-points. A standardization of the evaluation techniques and the reported parameters could be achieved for example by using the gold-standard micro-CT, reporting a minimum parameter set of BV/TV, Tb.Th, Tb.Sp, and Tb.N of the subchondral trabecular bone (as recommended in the classical paper of Bouxsein et al.) [8], together with subchondral bone plate thickness and osteophytes. These parameters can be reliably and accurately detected also in patients with clinical CT [40], allowing for a direct comparisons with the animal models. Still, only 22% of all examined studies report these 4 recommended trabecular parameters, and only 9% of all studies present the extended parameter set including subchondral bone plate thickness and osteophytes.

**Table 7:** Recommendations and considerations for future studies examining OA development in the subchondral bone following meniscal injury

Aspect to consider	Future studies	Explanation
Controls	Add normal/sham controls	Appropriate controls were missing in several publications, and these studies had to be excluded from the present review
Surgical protocol	Clear and detailed description needed	Description of surgical protocols was often insufficient to clearly identify the applied method of OA induction and relate it to the clinical problem
Longitudinal studies	Investigate early, mid, late time points	To compare OA progression in different surgical models and animal species vs. human OA, longitudinal studies with multiple time points are needed
Selection of volumes of interests	Separate the subchondral bone plate from the subarticular spongiosa	Bone microstructural alterations may be opposite direction in the two subchondral bone regions, thus separate VOI selection is crucial
Gold-standard evaluation technique	Perform micro-CT	Micro-CT is capable of accurate 3D evaluation of the subchondral bone in high spatial resolution in contrast to 2D methods such as histology
Minimum set of micro-CT parameters	Evaluation of subarticular spongiosa: BV/TV, Tb.Th, Tb.Sp, and Tb.N	Recommended [8] for a comparable characterization of the subchondral bone across different studies and models
Extended set of micro-CT parameters	Evaluation of subchondral bone plate: thickness and osteophytes (in addition to the minimum set)	Additionally to the minimum set of subarticular spongiosa parameters recommended by Bouxsein et al. [8], these additional subchondral bone plate parameters give a more comprehensive view of the entire subchondral bone

*2D* 2-dimensional; *3D* 3-dimensional; *BV/TV* percent bone volume; *micro-CT* microcomputed-tomography; *OA* osteoarthritis; *Tb.N* trabecular number; *Tb.Sp* trabecular separation; *Tb.Th* trabecular thickness; *VOI* volume of interest

Definition of analysis volumes of interests (VOI) also needs standardization, by constantly separating the subchondral bone plate from the trabecular bone VOIs. Yet, many studies reported them together, even though separation is possible in all examined species [39]. This appears especially important because simultaneous different direction of numerical changes in the two bone regions, observed commonly in various models (e.g. an increased BV/TV in the subchondral bone plate and decreased BV/TV in the subchondral trabecular bone), would result in apparently unchanged total subchondral bone parameters, complicating to reveal existing structural alterations and possibly resulting in false conclusions.

In early human and late large animal OA related to traumatic meniscal injuries, structural subchondral data are largely absent that could provide crucial information about the temporal trajectory of changes. As many parameters decrease in early OA below normal, and increase above it in late OA (or vice versa), a direct comparison of the results is not feasible without a clear definition of “early”, “mid-term”, and “late” OA stages within each species. Depending on OA stage, an increase, decrease or no difference of a certain parameter vs. normal is also possible and may reliably mirror the actual disease stage. Combining multiple microstructural parameters may identify a phenotypical fingerprint of each stage of the disease. Thus, detailed longitudinal studies with identical surgical protocols, multiple (early, mid, late) time points, appropriate normal/sham controls, and reliable and exhaustive structural analyses are required in all species.

Limitations include the absence of large animal microstructural data on late OA, complicating the comparability with humans. They are required in the future. High quality longitudinal studies revealing subchondral bone microstructural changes following meniscal injuries are unavailable, but needed to determine which animal model species represents best the human condition most faithfully. By using in vivo micro-CT or multiple termination time points, comparable longitudinal animal data could also be collected. While the meniscus tear is traumatic in nearly all animal models, the more common clinical situation of degenerative tears needs more attention [30]. However, degenerative lesions of the meniscus will be difficult to imitate in animal models.

In sum, changes in the subarticular spongiosa have a short temporal priority over those of the subchondral bone plate. These early alterations involve a degradation of the trabecular elements, reduction of their number, loss of bone volume and reduced mineralization. Soon thereafter, the subchondral bone plate becomes sclerotic; its porosity first increases, then decreases. The specific human topographical pattern of a thinner subchondral bone plate in the region below both menisci is present solely in the larger species (partly in rabbits), but absent in rodents, an important fact to consider when designing animal studies examining subchondral consequences of meniscus damage. Large animal models are capable of providing high topographical detail, suggesting that they may represent suitable study systems reflecting the clinical complexities. Future studies need to assess the subchondral bone in a standardized fashion. Comparative longitudinal studies investigating its microstructure in early, mid-term, and late stages with appropriate normal controls in all larger animal species will allow addressing clinically relevant questions about fundamental determinants of subchondral bone remodeling in knee OA caused by meniscal injuries.

## Data Availability

All data associated with this study are available in the main text.
